# Syphilitic Milkmen

**Published:** 1879-02

**Authors:** 


SYPHILITIC MILKMEN.—An English writer, Mr. George Gaskoin, has lately directed attention to the possible infection of milk through that very common form of secondary syphilis, psoriasis palmaria, on the hands of the men engaged in milking. He remarks: "Although the subject is of an unpleasant character, I think it would be possible to insist on a great circumspection in the choice of men who have this duty to perform. If the 'neat-handed Phillis' is to be discarded, which I cannot but think of with regret, we ought to be very particular in the class of men employed. Thus, for instance, it is obvious that discharged soldiers and seamen, in whom the probability is not small that they may have had syphilis, are not the people we should make choice of for milking our cows; but, in short, whoever has had this complaint in any degree, or at any period of his existence, is unsuited for so delicate a duty." This warning is very applicable to the daries around Philadelphia and New York, where the "milk maids" are usually boys and men.

